# Association Between Back Muscle Strength and Proprioception or Mechanoreceptor Control Strategy in Postural Balance in Elderly Adults with Lumbar Spondylosis

**DOI:** 10.3390/healthcare8010058

**Published:** 2020-03-10

**Authors:** Tadashi Ito, Yoshihito Sakai, Yohei Ito, Kazunori Yamazaki, Yoshifumi Morita

**Affiliations:** 1 Three-Dimensional Motion Analysis Room, Aichi Prefectural Mikawa Aoitori Medical and Rehabilitation Center for Developmental Disabilities, Okazaki 444-0002, Japan; 2 Department of Physical Therapy, Graduate School of Medicine, Nagoya University, Nagoya 461-8673, Japan; 3 Department of Orthopedic Surgery, National Center for Geriatrics and Gerontology, Obu 474-8501, Japan; jsakai@ncgg.go.jp; 4 Department of Electrical and Mechanical Engineering, Graduate School of Engineering, Nagoya Institute of Technology, Nagoya 466-8555, Japan; y.ito.359@nitech.jp (Y.I.); morita@nitech.ac.jp (Y.M.); 5 Faculty of Clinical Engineering, School of Health Sciences, Fujita Health University, Toyoake 470-1192, Japan; ymzkk@fujita-hu.ac.jp

**Keywords:** back muscle strength, mechanoreceptor, postural control, postural strategy, proprioception

## Abstract

This study aimed to investigate the relationship between back muscle strength and proprioception or mechanoreceptor control strategies used for postural balance in elderly adults with lumbar spondylosis. The displacement of the center of pressure (COP) excursion was determined in 24 elderly adults with lumbar spondylosis and 24 healthy young adults while the participants were standing upright on a balance board with their eyes closed. Vibratory stimulations of 30, 60, and 240 Hz were applied to the gastrocnemius (GS) and lumbar multifidus (LM) muscles to evaluate the effect of different proprioceptive signals on postural control. Back muscle strength was evaluated. Spearman’s rank correlation analysis was performed to determine the relationship between back muscle strength and significant COP excursion. Compared with young adults, elderly adults with lumbar spondylosis showed an increase in COP excursion displacement when a vibratory stimulation of 240 Hz was applied to the GS (*P* = 0.002) and LM muscles (*P* < 0.001). LM stimulation at 240 Hz was significantly associated with back muscle strength (*P* = 0.038). Postural control assessment with 240-Hz mechanoreceptor stimulation of the trunk could be a good indicator of postural instability due to over-dependence on mechanoreceptors and back muscle weakness in elderly adults with lumbar spondylosis.

## 1. Introduction

Postural control is essential for performing daily living activities in both elderly and young adults. To detect motion and adjust voluntary and reflexive muscle responses, postural control differentiates sensory information from visual, vestibular, and proprioception stimulation [1]. Healthy individuals normally maintain postural control using a multisegmental control strategy [2,3]. Bryan et al. [4] suggested that the only way to measure proprioception is through microneurography of afferent sensory signals. However, a previous analysis of the center of pressure (COP) excursion motion showed that elderly adults with spinal column stenosis and spondylitis deformans could not initiate or control a hip strategy with mechanoreceptor inputs [5]. Researchers have reported that elderly individuals control postural balance using a more rigid strategy that involves the ankle [6,7]. Putative impairments include pain, the decline in postural balance and back muscle function, and an altered proprioception system [8,9,10]. Therefore, the postural control of elderly adults with lumbar spondylosis may be negatively influenced by weak back muscles, which could, in turn, result in over-dependence on proprioception or mechanoreceptors [8,9,10]. Proprioception plays an integral role in modifying internal models used in the control of feedforward [11]. Moreover, Bryan et al. [12] suggested that muscle activity increases before the loading of joints during feedforward. Theoretically, trunk proprioception is essential for the accurate modulation and activation of muscles and joints to provide adequate neuromuscular control of the trunk position and joint movement and, therefore, postural control [12,13,14]. In cases of poor proprioception or mechanoreceptor inputs, postural control could only be maintained with sufficient muscle strength to compensate for the decrease in the accuracy of modulation and activation of muscles and joints [12,13,14]. Butler et al. [13] reported that the relative failure of postural control in proprioception related to muscle weakness indicates a functional link between sensory and contractile muscular processes. Moreover, van der Esch et al. [14] showed that the absence of adequate motor control could be associated with poor proprioceptive inputs and that muscle weakness decreases functional ability. This implies that postural control may be more affected by both weak muscle strength and proprioceptive or mechanoreceptor inaccuracy. It is reasonable to postulate that some proprioceptive and mechanoreceptor signals may be derived from the trunk muscles and that these may be altered by back muscle strength. Furthermore, back muscle weakness may affect the postural strategy for the proprioceptive or mechanoreceptor signals of the trunk.

The relationship between back muscle strength and the adaptive proprioception and mechanoreceptor responses to changing vibratory stimulations has not yet been clearly established. Local vibration, which is known to be a strong stimulus for Meissner corpuscles, muscle spindles, and Vater-Pacini corpuscles, has been used to assess the role of proprioception or mechanoreceptors in postural control [5,6,7,8,9,10,15,16,17,18,19,20,21,22]. Investigating the specific role of proprioception and mechanoreceptors during stimulation is essential to gain insight into the variability of postural control strategies and the possible relationship between impaired proprioception or mechanoreceptors and back muscle strength. To the best of our knowledge, no previous studies have investigated the association between back muscle strength and vibration stimulation in elderly adults with lumbar spondylosis. Therefore, this study aimed to investigate the relationship between back muscle strength and proprioception or mechanoreceptor control strategies used for postural balance in elderly adults with lumbar spondylosis.

## 2. Materials and Methods 

### 2.1. Participants

In this study, 24 male and 24 female community-dwelling adults from Japan participated between October 2013 and January 2016. Moreover, we conducted a retrospective analysis involving community-dwelling adults. Written informed consent was obtained from all participants before inclusion, and the Ethics Committee of the National Center for Geriatrics and Gerontology approved this study (Institutional Review Board approval number: 586). All investigations were conducted according to the principles outlined in the Declaration of Helsinki. 

Twenty-four elderly adults were recruited (age >65 years (range 65–85 years, average 73.7 ± 4.4 years); female = 12, male = 12). The study participants were patients with spinal column stenosis and spondylitis deformans who presented for conservative treatment of their symptoms. The diagnosis of lumbar spondylosis was confirmed using L1/2 to L4/5 area magnetic resonance imaging by a spine surgeon. Participants had no history of disabling low back pain (LBP) of more than 3 months or arthralgia and did not require assistance in maintaining a standing posture. The diagnosis of LBP was confirmed by a spine surgeon. Moreover, 24 healthy young adults were included as the control group (age >18 years (age range 19–24 years, average 21.7 ± 1.2 years); female = 12, male = 12) (Table 1). None of the participants required daily living activity assistance. Participants with the following characteristics were excluded: balance disorders, vestibular function disorders, spinal compression fracture, spinal cord tumor, spinal infection, paralysis, ataxia, neurological disorders, and/or a history of spinal surgery.

### 2.2. Back Muscle Strength Assessment

**Figure 1:**
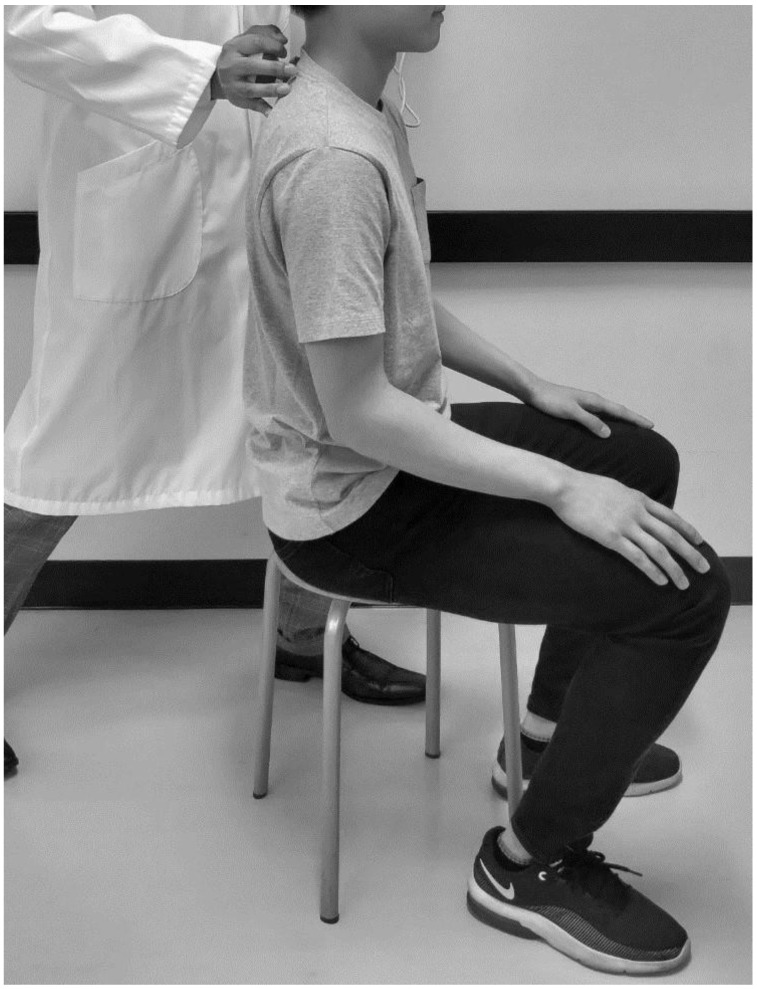
Experimental setup. Maximum isometric extension strength of the trunk muscle while in the sitting position with a 0° lumbar extension. Written informed consent was obtained from the participants before the publication of the image.

Back muscle strength was determined based on the maximum isometric extension strength of the trunk muscle in the sitting position with 0° trunk extension using a digital handheld dynamometer (Isoforce GT-300, 310; OG GIKEN Co., Ltd., Okayama, Japan). The mean back muscle strength value of two trials was determined and corrected for body weight (back muscle strength/weight: N/kg) (Figure 1).

### 2.3. Muscle Vibration

**Figure 2:**
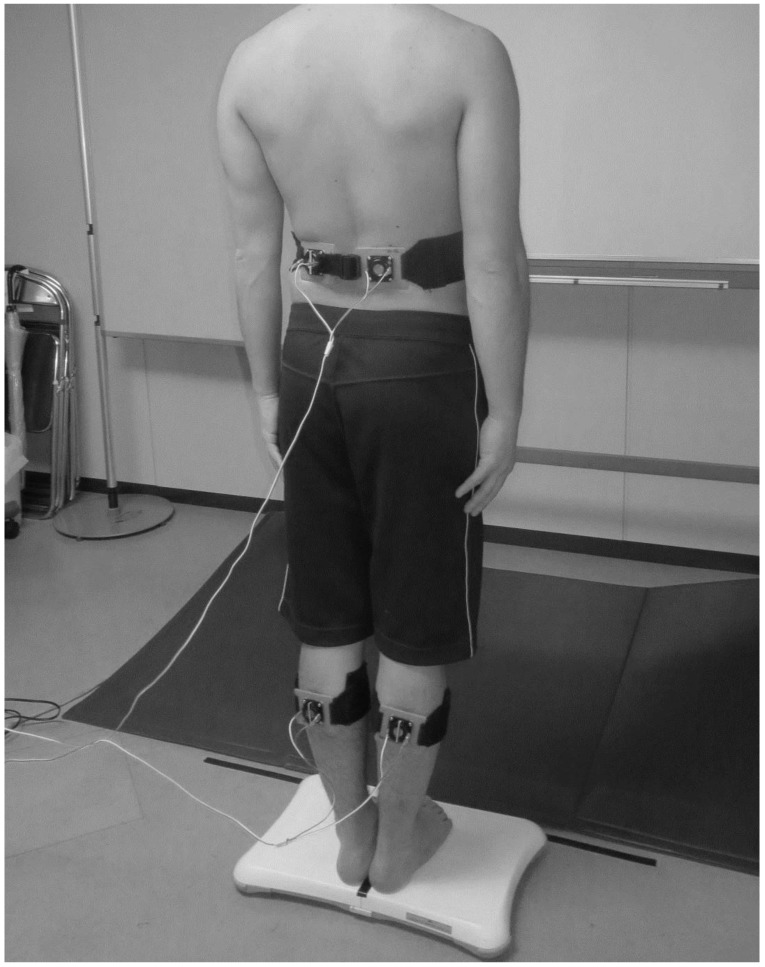
Experimental setup. Lumbar multifidus muscle and gastrocnemius muscle vibration during upright standing on a balance board.

COP excursion was recorded using a balance board (Wii; Nintendo Co., Ltd., Kyoto, Japan) [5,16,17,18,23,24,25]. Balance board data were acquired using a sampling frequency of 100 Hz and calculated using MATLAB (MathWorks, Inc., Natick, MA, USA). Balance board data were filtered using a low-pass filter with a cutoff frequency of 20 Hz. Vibratory stimulation was applied alternately by fixing four vibrators from the vibration device onto the participant’s gastrocnemius (GS) and lumbar multifidus (LM) muscles. The participants stood barefoot on the balance board with their feet together and their eyes closed. They were instructed to remain still and relax while their arms hung loosely at their sides (Figure 2). Previous studies of local vibratory stimulations reported an optimal response of the Meissner corpuscle, muscle spindles, and Vater-Pacini corpuscles to vibration frequencies of 5–40, 60, and 200–250 Hz, respectively [8,26,27]. Each subject’s COP excursion was measured under six conditions, that is, two muscles × three frequencies of vibratory stimulation: (1) 30 Hz on GS, (2) 30 Hz on LM, (3) 60 Hz on GS, (4) 60 Hz on LM, (5) 240 Hz on GS, and (6) 240 Hz on LM. The measurement time was 30 s, which was divided into two intervals of 15 s each [5,16,17,18]. Vibratory stimulation was applied to the participants during the last 15 s. We labeled the first 15 s as “Pre” and the last 15 s as “During.” The participants rested on a chair for 60 s between measurements.

Mean COP excursion displacement was defined as follows: ΔY = Y (During) − Y (Pre). Further, the COP excursion of the Y-coordinate data was calculated by the root mean square values of the COP excursion displacements in the postural stability trials. 

### 2.4. Low Back Pain Assessment

The pain was assessed using the visual analog scale (VAS) (0–10) [5].

### 2.5. Statistical Analysis

Normal distributions were confirmed using the Shapiro–Wilk test. Data of elderly and healthy young adults were compared using the Mann–Whitney U test or independent samples *t*-tests. A Bonferroni correction was used to compare different COP excursions during each vibratory stimulation frequency for GS between the elderly and young adults, with an α threshold of 0.016 (0.05/3). Further, for the comparison of postural strategies between elderly adults with lumbar spondylosis and healthy young adults during vibratory stimulation for LM, the Bonferroni-corrected significance level was set at 0.016 (0.05/3). Moreover, Spearman’s rank correlation analysis was performed to determine the relationship between back muscle strength and significant COP excursion. The participants were divided into the following two groups: elderly individuals and healthy young adults. Statistical significance for Spearman’s rank correlation analysis was set (a priori) at *P* < 0.05. All analyses were performed using commercially available software (IBM SPSS statistics software version 23; SPSS Inc., Chicago, IL, USA).

## 3. Results

When muscle vibrations of 30 and 60 Hz were applied to the GS and LM muscles, there were no significant differences in COP excursion between the two groups. There were no significant differences in back muscle strength between the two groups. However, compared to healthy young adults, elderly adults swayed a lot when a muscle vibration of 240 Hz was applied to the GS and LM muscles (Table 1 and Table 2). Elderly adults showed a higher VAS compared to healthy young adults (Table 1). Results of the Spearman’s rank correlation analysis showed that in elderly adults, the response of the GS to 240-Hz vibration was not significantly correlated with back muscle strength (r = −0.226; *P* = 0.288), whereas the LM (240 Hz) had a moderately negative correlation with back muscle strength (r = −0.425; *P* = 0.038). No correlations between back muscle strength and COP excursion displacements during GS and LM vibrations of 240 Hz were noted in healthy young adults (GS: r = −0.078; *P* = 0.716; LM: r = −0.034; *P* = 0.875).

## 4. Discussion

Our main finding was that there tends to be over-dependence on the mechanoreceptor input of the LM (240 Hz stimulation) during standing on a slightly unstable surface because of back muscle weakness, which could increase the risk of postural instability in elderly adults with lumbar spondylosis. Shakoor et al. [28] reported that decreased proprioception is significantly associated with decreased muscle strength of the hip limb. Furthermore, they reported that poor proprioception could lead to impaired sensory control of the limbs during periods of movement and when there is increased dynamic joint load. Previous studies have shown that elderly patients with LBP depend on proprioceptive inputs of the Vater-Pacini corpuscles [5,17], which may imply that the postural strategy adopted by elderly adults with lumbar spondylosis depends on the mechanoreceptors of the Vater-Pacini corpuscles in the LM and GS muscles. Other studies reported that proprioception and muscle spindles in the trunk decrease in both young and elderly adults with LBP [6,7,8,9,10,15,16,19,20,21]. However, in our study, no significant differences in COP excursion with 30- or 60-Hz stimulation to the GS or LM were found between the elderly patients with lumbar spondylosis and healthy young adults. Moreover, while the direct relationships between Meissner corpuscles, muscle spindles, Vater-Pacini corpuscles, and back muscles among healthy young adults are weak, back muscles have an indirect relationship with Vater-Pacini corpuscles during LM stimulation among elderly adults with lumbar spondylosis. Differences in proprioception and mechanoreceptor input as a result of variations in frequency stimulation may help to understand the association between postural control and back muscle strength. Thus, further studies on proprioception and mechanoreceptor input are warranted. 

Furthermore, a novel finding of our study was the significant relationship between postural control of the LM muscle at 240 Hz and back muscle strength in elderly adults with lumbar spondylosis. Previous studies reported how proprioception impairments could lead to a decline in muscle strength and emphasized the associations among determinants that influence proprioception control [29,30,31]. Our data suggested that the COP excursion of the LM at the Vater-Pacini corpuscles decreased as back muscle strength increased. Thus, the postural stability of the trunk in elderly adults with lumbar spondylosis could be associated with strong back muscles and does not depend on postural control by mechanoreceptor inputs. Postural control alterations could be explained by neuromuscular factors, including a decrease in cutaneous sensitivity or decreased capacity to generate and control muscle strength [32,33]. In another study, van der Esch et al. [14] reported that the combination of poor proprioception and muscle weakness has more functional limitations than poor proprioception alone. Hence, although the results in our study could be related to alterations in the position sense of Vater-Pacini corpuscles in the trunk and muscle strength, our findings suggested that weak back muscles might depend on the afferent feedback from cutaneous receptors, which might, in turn, lead to dependence on the position sense of Vater-Pacini corpuscles in the trunk. 

This study has a few limitations. First, despite the association between back muscle strength and the mechanoreceptor system (which was identified through muscle vibration), whether the mechanoreceptor control changes of single-joint movements based on mechanoreceptor perception are associated with back muscle weakness or changes in sensory processing during muscle activity or a combination of both remains unclear. Second, kinematic data to further support the findings of this study are lacking; we have no records of gluteus maximus or gastroc-soleus muscle activity. To better reflect postural control, the postural task designed to evaluate proprioception or mechanoreceptor input should include providing records of electrolysis of the muscle activities of the gluteus maximus and gastroc-soleus muscles. In addition, gluteus maximus activity could give insight into the postural strategy observed during unexpected or more difficult perturbation conditions. Moreover, both gluteus maximus and gastroc-soleus muscle activities could provide essential information that may help to maintain postural stability. Finally, only static standing postural control was examined in elderly adults without LBP and in healthy young adults. Proprioceptive data in those with more non-specific, recurrent, acute, and juvenile LBP could provide insight regarding the role of proprioception and mechanoreceptor input in postural strategy. Moreover, further assessment of the proprioceptive and mechanoreceptor inputs during vibratory stimulation may contribute to the rehabilitation of elderly adults with postural difficulties. Further longitudinal studies are required to validate our findings.

## 5. Conclusions

The results of the present study showed that during postural perturbation, back muscle weakness had a negative effect on a postural strategy involving mechanoreceptor input in elderly adults with lumbar spondylosis. Moreover, this study found that the proprioceptive and mechanoreceptor inputs of the lower leg and trunk of elderly adults with lumbar spondylosis were dependent on the Vater-Pacini corpuscles rather than on the Meissner corpuscles and muscle spindles. Over-dependence on the mechanoreceptor input of the LM postural strategy while standing on a slightly unstable surface as well as weak back muscle strength could increase the risk of postural instability in elderly adults with lumbar spondylosis. Our findings showed that postural control assessment with 240-Hz mechanoreceptor stimulation of the trunk could be a good indicator of postural instability due to the over-dependence on mechanoreceptors and weak back muscle strength in elderly adults with lumbar spondylosis.

## Figures and Tables

**Table 1:** Demographic characteristics of the participants.

Variables	Elderly Adults with Lumbar Spondylosis (*n* = 24)	Healthy Young Adults (*n* = 24)	*P*
Age (years)	73.5 (65–85)	22 (19–24)	0.001
Sex (men)	12 (50)	12 (50)	1.000
Height (cm)	158.6 ± 9.6	165.6 ± 6.3	0.004
Weight (kg)	59.6 ± 10.6	55.6 ± 8.0	0.151
BMI (kg/m^2^)	23.7 (16.5–30.9)	20.3 (17.1–26.7)	0.001
VAS (cm)	0.5 (0–5)	0 (0–0)	0.001
Back muscle strength (N/kg)	2.9 ± 0.6	3.2 ± 0.6	0.110

Data are presented as the mean ± standard deviation or median value (minimum and maximum values) or n (%). The *P*-value for age was calculated using the Mann–Whitney U test. Other *P*-values were generated using independent samples t-tests or Chi-square tests. BMI, body mass index; VAS, visual analog scale.

**Table 2:** Mean displacements of the center of pressure (COP) during the vibration trials for elderly adults with lumbar spondylosis and healthy young adults while standing on a balance board.

Variables	Elderly Adults with Lumbar Spondylosis (*n* = 24)	Healthy Young Adults (*n* = 24)	*P*
GS with 30 Hz (cm)	0.82 (0.43–1.73)	0.67 (0.27–1.3)	0.030
GS with 60 Hz (cm)	0.84 (0.43–1.67)	0.69 (0.18–1.57)	0.274
GS with 240 Hz (cm)	0.87 (0.45–1.62)	0.52 (0.28–1.57)	0.002
LM with 30 Hz (cm)	0.63 (0.44–1.2)	0.61 (0.28–1.28)	0.578
LM with 60 Hz (cm)	0.70 (0.46–1.43)	0.64 (0.32–1.13)	0.080
LM with 240 Hz (cm)	0.74 ± 0.21	0.56 ± 0.15	0.001

Data are presented as the mean ± standard deviation or median value (minimum and maximum values). The *P*-value for LM with 240 Hz was generated using independent samples t-tests. Other *P*-values were calculated using the Mann–Whitney U test. GS, gastrocnemius; LM, lumbar multifidus muscles.
